# Single-Atom Underpotential Deposition at Specific Sites of N-Doped Graphene for Hydrogen Evolution Reaction Electrocatalysis

**DOI:** 10.3390/ma17205082

**Published:** 2024-10-18

**Authors:** Haofei Wu, Qiwen Zhang, Shufen Chu, Hao Du, Yanyue Wang, Pan Liu

**Affiliations:** 1 State Key Laboratory of Metal Matrix Composites, School of Materials Science and Engineering, Shanghai Jiao Tong University, Shanghai 200240, China; sjtumsewu@sjtu.edu.cn (H.W.);; 2 Shanghai Key Laboratory of Advanced High-Temperature Materials and Precision Forming, Shanghai Jiao Tong University, Shanghai 200240, China; 3 Shanghai Jiao Tong University—JA Solar New Energy Materials Joint Research Center, Shanghai 200240, China; 4 National Engineering Research Center of Light Alloy Net Forming and State Key Laboratory of Metal Matrix Composite, Shanghai Jiao Tong University, Shanghai 200240, China; 5 JA Solar Technology Co., Ltd., Beijing 100160, China

**Keywords:** underpotential deposition, single-atom catalyst, hydrogen evolution reaction, N-doped graphene

## Abstract

Single-atom catalysts (SACs) have the advantages of good active site uniformity, high atom utilization, and high catalytic activity. However, the study of its controllable synthesis still needs to be thoroughly investigated. In this paper, we deposited Cu SAs on nanoporous N-doped graphene by underpotential deposition and further obtained a Pt SAC by a galvanic process. Electrochemical and spectroscopic analyses showed that the pyridine-like N defect sites are the specific sites for the underpotential-deposited SAs. The obtained Pt SAC exhibits a good activity in a hydrogen evolution reaction with a turnover frequency of 25.1 s^−1^. This work reveals the specific sites of UPD of SAs on N-doped graphene and their potential applications in HERs, which provides a new idea for the design and synthesis of SACs.

## 1. Introduction

Worldwide interest in H_2_ as a clean chemical energy source to replace fossil fuels and avoid CO_2_ emissions is increasing [1,2,3]. The electrocatalytic hydrogen evolution reaction (HER) based on renewable electricity is a promising, safe, scalable, low-cost, green method of producing H_2_ [4,5,6]. The noble metal Pt is considered the most efficient catalyst for use in the HER [7,8,9]. However, owing to its high cost, further consideration is required to maximize the efficiency of precious metal utilization and improve the activity of the catalyst by modulating its electronic structure [10,11,12].

Carbon is widely used as a multifunctional material in numerous fields. In the field of hydrogen energy, due to its good electrical conductivity, high chemical stability, and high specific surface area, it can not only be used as a hydrogen storage material [13,14] but can also widely be used as a substrate material for loaded catalysts. Loaded nanocatalysts are immobilized on supports, and the electronic structures of the active sites of the catalysts can be efficiently regulated via strong metal–support interactions, which are described as electronic metal–support interactions (EMSIs) [15,16,17]. Single-atom catalysts (SACs) minimize the catalytic structures with well-defined active sites. Their homogeneous atomic coordination environments render them as ideal simplified model systems for use in studying catalytic reaction mechanisms [18,19,20]. Strong EMSIs not only stabilize single-atom metals by forming thermodynamically favorable metal–support bonds but also modulate the electronic structures of single-atom metals and their catalytic activities via electron transfer, leading to charge redistribution [21,22,23].

Now, commonly used synthesis methods for SACs often require the usage of high energies to drive the formation of SAs. For example, Shah et al. synthesized a Co SAC on RuO_2_ spheres by hydrothermal reactions coupled with calcination for HER and OER (oxygen evolution reaction) bifunctional catalysis [24]. Jin et al. prepared K_2_PtCl_4_@NC-M SAC using high-energy ball milling at 400 rpm for 20 min, which showed a 17-fold enhanced mass activity over commercial catalysts [25]. Qu et al. used defects in defective graphene (DG) to trap Pt atoms at high temperatures and gradually converted bulk Pt into Pt Sas; the prepared Pt SACs/DG exhibited high HER activity [26]. Underpotential deposition is a mild typical surface self-limiting reaction that has been used for the growth of metal single-atom layers [27]. Since UPD tends to obtain continuous layers due to the uniform distribution of the substrate surface work function, Shi et al. proposed that single layers can be further upgraded to single atoms (SAs) by using materials with non-uniformly distributed Lewis base sites as substrates for underpotential deposition [28]. Transition metal disulfide compounds and carbon-based materials have been shown to be supports that can be used for the underpotential deposition of single atoms of copper, but the underpotential deposition-specific sites have been considered to be the S, Se, and O sites in previous studies, and studies on N sites as underpotential deposition sites are not yet understood [28,29,30].

In this work, we prepared a Pt SAC on nanoporous nitrogen-doped graphene (np-NG/Pt) by UPD and a galvanic process. Compared to conventional methods, the preparation of single atoms using UPD is carried out under milder conditions and is more energy efficient. Detailed spectroscopic and electrochemical data indicate that among the different kinds of N defect sites in N-doped graphene, the pyridine-like nitrogen defects are the specific sites for the formation of Cu SAs by UPD. The Pt SACs further formed by the galvanic process replacing the Cu SAs show good activity in an acidic HER.

## 2. Materials and Methods

### 2.1. Synthesis of Nanoporous Nitrogen-Doped Graphene (np-NG)

The fabrication of np-NG has been reported in detail in previous works [31,32]. Firstly, a Ni_30_Mn_70_ (atomic ratio) ingot, obtained by an arc-melting method, was cold-rolled into Ni_30_Mn_70_ sheets with a thickness of 150 μm. The Ni_30_Mn_70_ sheets were chemically dealloyed in a 1.0 M (NH_4_)_2_SO_4_ aqueous solution at 50 °C for 5 h, then thoroughly rinsed with deionized water and ethanol and dried in a vacuum oven at room temperature to obtain nanoporous Ni templates.

The np-Ni template was loaded into a quartz tube (φ50 × φ50 × 1400 mm) of a three-heating zone furnace, and np-NG was fabricated on the np-Ni template by a chemical vapor deposition (CVD) process. Melamine was used as the carbon and nitrogen sources for growing np-NG. A total of ~0.25 g of melamine powder and the np-Ni sheet were loaded into porcelain boats and placed in the central heating zone and the adjacent downstream heating zone, respectively. The downstream heating zone was first heated from room temperature to 800 °C within 30 min to anneal nanoporous Ni under the mixed gas atmosphere of Ar (200 sccm) and H2 (200 sccm). Meanwhile, the central heating zone was gradually ramped to 200 °C within 30 min. During the 5 min pre-reduction annealing, melamine powders were further heated to 300 °C. When the temperature of the central heating zone approaches 300 °C, melamine powders start to evaporate and form a continuous graphene film on the Ni substrate. The furnace was opened immediately after 7 min of graphene growth and cooled to room temperature using a fan to obtain np-Ni/NG. The samples without N doping were prepared in a similar way as np-Ni/NG, with the gas atmosphere and annealing temperature unchanged, and the precursor was changed to toluene. When np-Ni was pre-reduced and annealed for 5 min, toluene gas was passed through the furnace and the reaction was carried out for 5 min at 800 °C, then the furnace was opened immediately and cooled down to room temperature using a fan to obtain the np-Ni/G.

The np-Ni/NG and np-Ni/G sheets were etched with a 2.0 M HCl solution containing 5 mM FeCl_3_ at 50 °C overnight to completely etch Ni and then rinsed five times with deionized water. Then, N-doped np-NG and non-N-doped np-G can be obtained by the freeze-drying technique. According to XPS, the N content is about 6.3%.

### 2.2. Synthesis of Cu SAC and Pt SAC

The Pt SAC was synthesized according to the previous report [22]. The np-NG was mixed with a 0.5% Nafion ethanol solution and sonicated to an ink with a concentration of 4 mg/mL. The φ5 mm glassy carbon (GC) electrodes were polished using alumina powder, cleaned in ethanol for a short time by ultrasonication, and dried at room temperature. A total of 5 µL of np-NG ink was dropped onto the glassy carbon electrode and dried under a hot lamp to make a working electrode. The preparation of single atoms on np-NG used an underpotential deposition (UPD) method, using a Ag/AgCl electrode (3.0 M KCl, E^θ^ = 0.21 V) as a reference electrode and a 1 × 1 cm^2^ Pt sheet as a counter electrode.

Firstly, Cu atoms were underpotentially deposited on the np-NG by controlling the potential at +0.10 V vs. Ag/AgCl for 120 s in a 0.1 M H_2_SO_4_ saturated Ar solution containing 2 mM CuSO_4_, and this sample was named np-NG/Cu. The np-NG/Cu sample was washed with deionized water and then transferred to an Ar-saturated solution of 50 mM H_2_SO_4_ containing 2 mM K_2_PtCl_4_ and allowed to stand for 10 min at open circuit potential to displace the Cu with Pt (II). The new sample was termed as np-NG/Pt.

### 2.3. Structure Characterizations

The morphologies of different samples were characterized by scanning electron microscope (SEM, 3D Versa, FEI, Hillsboro, OR, USA) at a 20 kV beam voltage. The chemical compositions of the samples were investigated by X-ray photoelectron spectroscopy (XPS) utilizing the AXIS UltraDLD XPS system (Kratos, Shimadzu, Kyoto, Japan). High-resolution TEM (HRTEM) images of all samples were obtained by JEOL ARM 200F (Tokyo, Japan) equipped with a spherical aberration corrector for the image-forming objective lens operating at a 200 kV acceleration voltage. The high-angle annular dark-field scanning transmission electron microscopy (HAADF-STEM) images were obtained by JEOL ARM 200F equipped with a spherical aberration corrector for the probe-forming lens and an energy-dispersive X-ray spectrometer (EDS). X-ray absorption fine structure (XAFS) measurements were conducted on the BL11B beamline at Shanghai Synchrotron Radiation Facility (SSRF), Shanghai, China.

### 2.4. Electrochemical Test

The electrochemical performance was tested in a three-electrode system with a 0.5 M H_2_SO_4_ electrolyte on a CHI 760E electrochemical workshop. A graphite electrode was used as the counter electrode and the Ag/AgCl electrode (3.0 M KCl, E^θ^ = 0.21 V) was used as the reference electrode. Different catalysts were loaded on the φ5 mm GC electrode as the working electrode. The loading amount of commercial 20% Pt/C or different np-NG catalysts were all 100 μg/cm^2^. The linear sweep voltammetry (LSV) curve was obtained at a scan rate of 5 mV/s with a 90% iR drop correction. EIS was carried out in the frequency range of 100 kHz to 0.01 Hz at the overpotentials of 50 mV in the 0.5 M H_2_SO_4_ electrolyte. Relative standard errors for all components were controlled to <10% in the EIS fitting.

Electrochemical active surface areas (ECSAs) of different Pt catalysts were obtained by calculating H underpotential adsorption (H_UPD_) by cyclic voltammetry tests in the range of −0.21 V~0.5 V (vs. Ag/AgCl) in the 0.5 M H_2_SO_4_ electrolyte. The equation for calculating the *ECSA* of *Pt* is shown in Equation (1).

(1) ECSA=SHv0.21(mC/cm2)·mPt

where *S_H_* is the integral area of the H_UPD_ peak, *v* is the sweep speed, and *m_Pt_* is the Pt loading mass.

### 2.5. Turnover Frequency (TOF) Calculations for the HER

The TOF calculation starts with the number of moles of active sites of the catalyst, *n*. For SACs, one atom can be considered as one active site, so *n* can be obtained from Equation (2).

(2) $$n=\frac{m}{M}$$

where *m* is the loading mass of a single atom and *M* is the molar mass of a single atomic element. For Pt nanoparticles, the number of catalytically active sites can be deduced from the ECSA since it has been obtained by H underpotential deposition; the *n* can be obtained from Equation (3).

(3) $$n=\frac{\rho \cdot ECSA\cdot m_{Pt}}{N_{A}}$$

where *ECSA* is the electrochemical active surface area; *N_A_* is the Avogadro constant (≈6.022 × 10^23^); *m_Pt_* is the Pt loading mass; and *ρ* is the number of active sites in a plane per unit area of Pt (1.637 × 10^15^ active atoms/cm^2^) [33,34].

The TOF value is calculated based on Equation (4).

(4) $$TOF=\frac{J\cdot A}{2\cdot F\cdot n}$$

where *A* is the geometric area of the φ5 GC electrode (0.196 cm^2^); F is the Faraday constant (96,485 C/mol); *n* is the number of moles of active materials that are loaded onto the electrode; and *J* is the is the current density. *J* can be categorized into two types; one is the exchange current density (*J*_0_) [35] and the other is the kinetic current density (*J_k_*) [36]. For the HER, which is less affected by the mass transfer, the measured current can be approximated to be the kinetic current, so here we choose to use the more intuitive *J_k_* to calculate the TOF.

## 3. Results and Discussion

**Figure 1:**
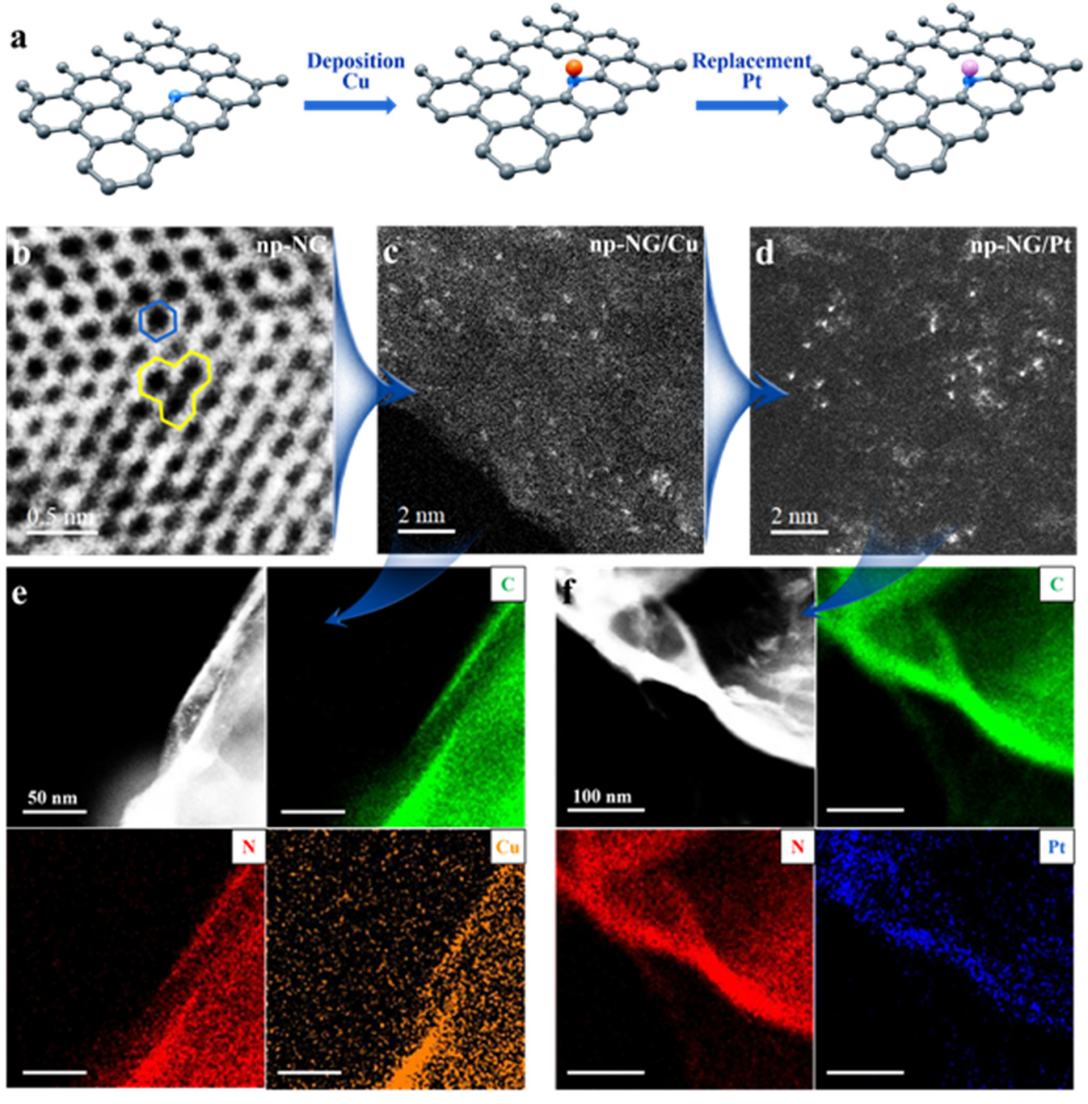
Single-atom design and characterization of the np-NG support. (**a**) The schematic of the UPD of single atoms. (**b**) HAADF-STEM image of defective carbon hexagon structure of np-NG. HAADF-STEM images of (**c**) np-NG/Cu and (**d**) np-NG/Pt. Corresponding elemental mapping of (**e**) np-NG/Cu and (**f**) np-NG/Pt.

Figure 1a shows a schematic of the UPD of single atoms. The np-NG was obtained by using nanoporous Ni as a template, and its biconnected porous structure enabled better self-support, a larger specific surface area, and better mass transfer channels as a reaction support (Figure S1). Compared to 2D graphene, the high curvature introduces more defects for np-NG that can be used as growth sites for single atoms, as shown in the aberration-corrected high-angle annular dark-field scanning transmission electron microscopy (HAADF-STEM) image in Figure 1b. The blue hexagon is a typical graphene six-membered ring structure and the yellow area is a defect that lacks a carbon atom and can be used as the location to hold metal atoms. To enable the self-terminating growth of single-atom Pt on the np-NG carriers, Cu atoms are first underpotentially deposited on np-NG (denoted np-NG/Cu, Figure 1c). The Cu atoms are then galvanically exchanged with Pt(II) to form the np-NG-supported single-atom Pt samples (denoted np-NG/Pt, Figure 1d). HAADF-STEM images show atomically dispersed metal atoms (bright spots) on the np-NG/Cu and np-NG/Pt. Surface-confined UPD enables the formation of energetically favorable metal–support bonds and automatically terminates the sequential formation of metal bonds [28]. Energy-dispersive X-ray spectroscopy elemental mapping reveals that the Cu and Pt atoms are homogeneously dispersed (Figure 1e,f).

**Figure 2:**
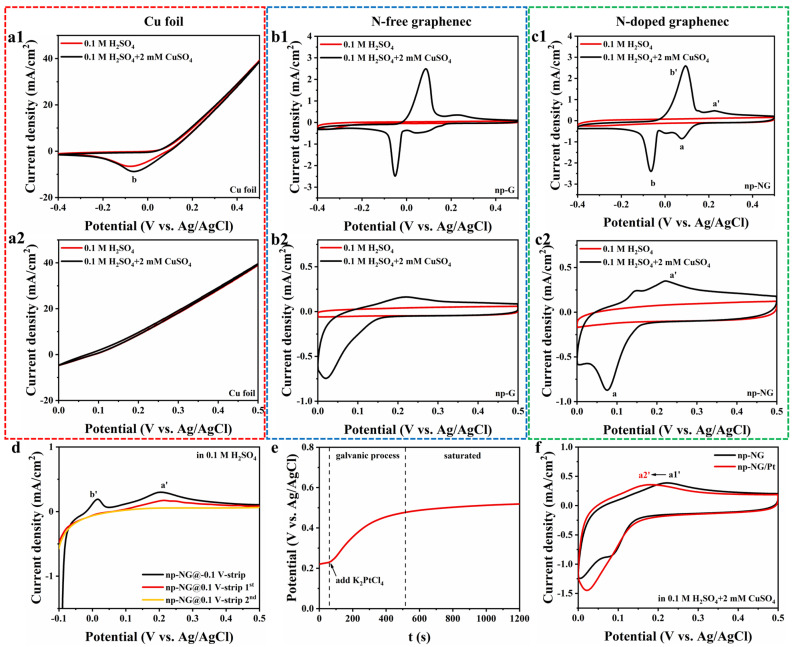
Electrochemical deposition of Cu on different samples. CV curves of Cu redox on (**a1**,**a2**) Cu foil, (**b1**,**b2**) np-G, and (**c1**,**c2**) np-NG samples over different potential windows. (**d**) LSV stripping curves of Cu deposited at different potentials. (**e**) Monitoring of the open circuit potential of the galvanic process. (**f**) CV curves of Cu redox on np-NG before and after loading Pt SAs.

The redox peaks of Cu within the different samples were compared within a wide potential window (−0.4~0 V) to exclude the influences of other factors. All the CV curves were obtained by sweeping from positive to negative potentials (cathodic sweep) and then from negative to positive potentials (anodic sweep). In the Cu foil sample, the reduction peak b is present irrespective of the presence of CuSO_4_, which may be due to the oxide layer on the surface [37]. However, it can be seen that in the electrolyte containing CuSO_4_, the intensity of the b peak is enhanced due to the superimposed reduction current of Cu^2+^. The Cu^2+^ reduction current occurs at a position between −0.1 and ~0 V, representing the bulk deposition peak b of Cu (Figure 2a1) [38]. In the cyclic voltammograms of the clean glassy carbon electrode (GC) and the GC with a drop of Nafion solution, only the bulk deposition peaks of Cu appear (Figure S2). No significant reduction reaction occurred within the 0~0.5 V voltage window (Figure 2a2). In contrast, in the cyclic voltammograms of the graphene samples, in addition to the bulk deposition peaks of Cu, reduction peaks are observed at more positive deposition potentials, suggesting strong interactions between Cu and graphene. However, in the cyclic voltammograms of graphene with and without N defects, a difference in the UPD of Cu is observed in Figure 2b1,c1. The intensities of the reduction peaks of UPD and the oxidation peaks upon dissolution when N defects are present (Figure 2c2, a_np-NG_′ peak, 0.35 mA/cm^2^@0.22 V) are significantly stronger than those of the np-G samples without N defects (Figure 2b2, a_np-G_′ peak, 0.16 mA/cm^2^@0.22 V). The presence of a weak UPD peak in the curve of np-G may be caused by O defects introduced during the etching of the Ni template. Therefore, N defects are considered as specific sites for the UPD of single Cu atoms on nanoporous graphene substrates.

The constant–potential deposition curves also indicate the difference between the two samples (Figure S3). The np-NG curve exhibits a clear UPD region, and the magnitude current gradually increases from −0.6 to 0 mA/cm^2^ as UPD proceeds, indicating that the Cu single atoms gradually occupy the surface as specific sites [30]. However, the np-G curve does not display a UPD region and the current remains at 0 mA/cm^2^ during the reaction, indicating that the number of sites that may be deposited on its surface is considerably less than that of np-NG. The voltage at which Cu bulk deposition occurs on np-NG was also chosen for potentiostatic deposition. The bulk deposition current is significantly larger than the UPD current, which coincides with the polarization curve. Stripping linear sweep voltammetry was performed after electrodeposition by transferring the samples to a Cu^2+^-free 0.1 M H_2_SO_4_ electrolyte to analyze the form of Cu deposition on np-NG. Double peaks of both bulk Cu and the Cu SA appeared in the bulk-deposited samples, while only a single peak of the Cu SA appeared in the UPD sample in the first run of stripping. It is shown that the Cu SA can be selectively obtained by controlling the deposition potential. In the second run of the Cu SA stripping curve, the Cu SA peak in the UPD sample had completely disappeared, indicating the single-layer structure. The change in the open circuit potential of np-NG/Cu before and after the addition of 5 mM K_2_PtCl_4_ in a 0.05 M H_2_SO_4_ solution was monitored. The addition of K_2_PtCl_4_ at 60 s, followed by a slow rise in open circuit potential, indicated that the displacement of Cu occurred on the sample. The open circuit potential gradually saturated after about 500 s, indicating that Cu was almost completely replaced [17]. CV curve analysis of the redox reaction of Cu on the np-NG/Pt SA samples showed that the intensity of the oxidation peak of Cu was almost unchanged compared to the pristine np-NG, but the position was negatively shifted from a1′ (0.22 V) to a2′ (0.2 V). This indicates that the original site has been covered by Pt and the change in the work function of the new site caused the change in redox potential.

**Figure 3:**
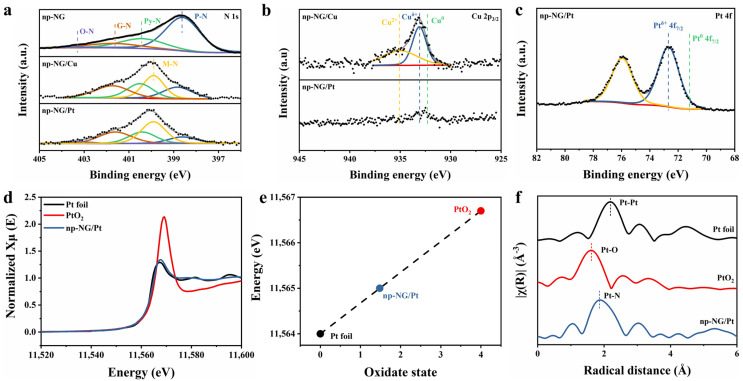
Structural characterization of different samples. XPS spectra of (**a**) N 1s, (**b**) Cu 2p, and (**c**) Pt 4f of different samples. (**d**) XANES and (**e**) correlation between the oxidation state and energy position. (**f**) Fourier transform EXAFS spectra of different Pt samples.

We investigated the chemical configurations and local coordination of different SA samples on the np-NG substrate using X-ray photoelectron (XPS) and X-ray absorption spectroscopy (XAS). Changes in N sites before and after UPD were evident by XPS. Based on the N 1s spectrum of pristine np-NG shown in Figure 3a, four different types of N are observed, namely pyridine-like (P-N, 398.6 eV, 62.4%), pyrrole-like (Py-N, 400.4 eV, 23.9%), graphitic (G-N, 401.6 eV, 12.8%), and oxidized (O-N, 403.3 eV, 0.9%) [39]. Remarkably, the percentage of P-N decreases significantly to 21.1% with the deposition of Cu SAs. A new metal–N peak (M-N) is observed at 399.9 eV, suggesting that the sites for the UPD of single atoms are mainly P-N sites, and the P-N sites are transformed into M-N sites after the generation of single atoms [40,41]. The N 1s spectrum of the sample after galvanic replacement are similar to that after the UPD of Cu, with a decrease in the P-N content and the appearance of the M-N peak, which suggests that interactions between the single atoms and the N sites still exist after replacement. When comparing the Cu 2p spectra before and after galvanic replacement (Figure 3b), a distinct Cu 2p peak is observed in the spectrum of np-NG/Cu. The Cu^δ+^ 2p_3/2_ peak is located at 933.08 eV between the peak positions for Cu(0) (932.60 eV) and Cu(II) (934.97 eV), indicating that 0 < δ(Cu) < 2, and electron transfer between Cu and N occurs. The Cu 2p signal almost disappears in the spectrum of the replaced sample, indicating that the Cu has been replaced. The 4f_7/2_ peak of Pt(0) is located at 71.2 eV and thus, the Pt 4f XP spectra shown in Figure 3c reveal that the Pt SAs occur in their oxidized states. Additionally, the Pt^δ+^ 4f_7/2_ peak in the spectrum of np-NG/Pt is located at 72.65 eV, which also indicates that 0 < δ(Pt) < 2 [42]. Hence, the strong electrostatic effects between the single atoms generated via galvanic replacement and the N defects on the support are still retained.

Based on X-ray absorption near-edge structure (XANES) spectroscopy, Pt L_3_-edge analysis (Figure 3d) shows that the white line intensities of np-NG/Pt are higher than those of Pt foils but lower than those of PtO_2_, suggesting that the Pt SAs display more unoccupied 5d orbitals compared to Pt(0) but do not reach valences of +4 [43]. The first-derivative XANES spectra shown in Figure S3 reveal that the valence states of both types of Pt SAs are between those of the reference Pt foil and PtO_2_. Based on the fitting of the specific oxidation states using the reference Pt foils and PtO_2_ (Figure 3e), the valence state of Pt in np-NG/Pt is +1.48, which is consistent with the XPS results. The local atom structure was further determined using extended X-ray absorption fine structure (EXAFS) analysis (Figure 1f). No Pt–Pt bonds are observed in np-NG/Pt, and a peak at 1.96 Å is observed instead. This peak may be attributed to Pt-N and Pt-C.

**Figure 4:**
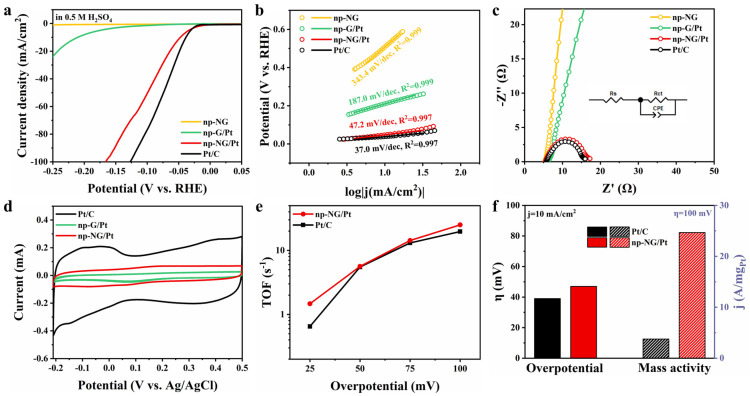
Electrochemical studies in 0.5 M H_2_SO_4_ electrolyte. (**a**) HER curves, (**b**) Tafel plots, (**c**) Nyquist plots, (**d**) ECSA curves, (**e**) TOF curves, and (**f**) overpotential (j = 10 mA/cm^2^) and mass activities (η = 100 mV) of different catalysts.

We have evaluated the HER performance of galvanically replaced np-NG/Pt SACs using a three-electrode system in the 0.5 M H_2_SO_4_ electrolyte. The Pt atom-loaded samples exhibit superior electrocatalytic HER activities compared to that of pristine np-NG. The different Pt catalysts exhibit different acidic HER activities in the order of commercial Pt/C (39 mV) > np-NG/Pt (47 mV) > np-G/Pt (211 mV) at a current density of 10 mA/cm^2^ (Figure 4a). The np-NG (508 mV) as substrates do not exhibit HER activity. The performance of np-NG/Pt slightly decreased after 1000 cycle tests (53 mV@10 mA/cm^2^), which may be due to the detachment of some poorly bound Pt SAs (Figure S4). To investigate the mechanisms of different samples in the HER under acidic conditions, we evaluated the catalytic kinetics using Tafel and Nyquist plots (Figure 4b,c). In all samples, the Tafel slope of np-NG/Pt (47.2 mV/dec) is close to that of commercial Pt/C (37.0 mV/dec) [44] and better than that of pristine np-NG (343.4 mV/dec), indicating that it exhibits good HER kinetics. Additionally, np-NG/Pt displays small charge transfer resistance (R_ct_) under acidic conditions (overpotential of 50 mV), and the order of R_ct_ measured at an overpotential of 50 mV is Pt/C (10.1 Ω) < np-NG/Pt (11.5 Ω) < np-NG (600.1 Ω), suggesting that electrons can be transferred rapidly across np-NG/Pt.

In contrast to commercial Pt/C electrodes, np-NG/Pt did not show significant characteristic redox peaks in the Pt-H adsorption/desorption region (Figure 4d). For bulk Pt materials, there is a strong association between the H_upd_ peak and the surface structure, which can be used as a fingerprint of the surface orientation [45]. This suggests that it is possible that firstly, the ultra-low Pt loading (2 wt.% by XPS) in the Pt SAC exceeds the detection limit of cyclic voltammetry and secondly, there are differences in the characteristic redox peaks of the Pt SAs with respect to the bulk material. We compared the TOF of the Pt SAC with that of commercial Pt/C. The number of active sites in commercial Pt/C was obtained based on the ECSA, whereas in the Pt SAC, it is approximated that each atom is an active site. The densities of active sites in Pt/C and np-NG/Pt were 2.02 × 10^−8^ mol/cm^2^ and 1.05 × 10^−8^ mol/cm^2^, respectively, indicating that the Pt atoms in np-NG/Pt were well utilized. And at a 100 mV overpotential, the TOF of np-NG/Pt is 25.1 s^−1^, which is higher than that of commercial Pt/C at 19.7 s^−1^ (Figure 4e), suggesting that np-NG/Pt has better intrinsic activity. Meanwhile, np-NG/Pt exhibits excellent mass activity due to the low noble metal loading, with a mass activity of 24.7 A/mg_Pt_ at a 100 mV overpotential, which is 6.7 times higher than that of commercial Pt/C. Several experimental and simulation results have shown that by lowering the oxidation state of Pt, the adsorption/desorption of H on the catalyst surface can be improved, thus enhancing the acidic HER performance of Pt [46,47,48,49]. This is consistent with the results we obtained in XPS and XAFS, where platinum has a lower valence and therefore exhibits good HER activity.

## 4. Conclusions

In conclusion, we developed a method to prepare Cu SAs on N-doped graphene by UPD. The conditions are milder and more energy efficient than conventional methods of preparing SAs. Fine XPS and XAFS were used for the identification of SAs and the corresponding anchored specific sites. It was found that among all the N sites, pyridine-like N defects are the specific sites for the UPD of Cu SAs. Furthermore, by the galvanic process method, Pt replaces Cu to obtain np-NG/Pt SAC. The EMSIs between the specific sites and the SAs were able to effectively immobilize the SAs and regulate the oxidation state of the Pt SACs, which showed good HER performance under acidic conditions. Although the loading of Pt SAs was so small that it could not even be detected by H absorption/desorption, it exhibited a TOF of 25.1 s^−1^ and mass activity of 126.5 mA/mg_Pt_ at 100 mV. This method provides a new understanding and design ideas for the bottom-up preparation of atomic structures on N-doped graphene.

## Data Availability

The original contributions presented in the study are included in the article/Supplementary Materials, further inquiries can be directed to the corresponding author.

## Supplementary Materials

The following supporting information can be downloaded at: https://www.mdpi.com/article/10.3390/ma17205082/s1, Figure S1: SEM images of np-Ni template (a) before and (b) after growing graphene and (c) np-NG after etching Ni template. (d) STEM and (e) TEM images of np-NG. (f) HRTEM image of the cross-section of np-NG; Figure S2: CV curves of different samples in 0.5 M H_2_SO_4_ containing 2 mM CuSO_4_; Figure S3: Chronoamperometry curves of the deposition of Cu at different potentials; Figure S4: The corresponding first-derivative XANES spectra of different Pt samples; Figure S5: Stability test of np-NG/Pt by potential cycling before and after 1000 cycles.
