# Supplementary material for: Single-Atom Underpotential Deposition at Specific Sites of N-Doped Graphene for Hydrogen Evolution Reaction Electrocatalysis
Source: Materials (Basel). 2024 Oct 18;17(20):5082. doi: 10.3390/ma17205082 (PMC11509329; doi:10.3390/ma17205082)
Supplement: Supplementary file 1 [file materials-17-05082-s001.zip › materials-3249224-supplementary.pdf]

Supporting Information

Single-Atom Underpotential Deposition at Specific Sites of N-Doped Graphene for Hydrogen Evolution Reaction Electrocatalysis

Haofei Wu [1,2,3], Qiwen Zhang [1,2,3], Shufen Chu [4], Hao Du [3,5], Yanyue Wang [3,5] and Pan Liu [1,2,3,*]

1 State Key Laboratory of Metal Matrix Composites, School of Materials Science and Engineering, Shanghai Jiao Tong University, Shanghai 200240, China; sjtumsewu@sjtu.edu.cn (H.W.)
2 Shanghai Key Laboratory of Advanced High-Temperature Materials and Precision Forming, Shanghai Jiao Tong University, Shanghai 200240, China
3 Shanghai Jiao Tong University—JA Solar New Energy Materials Joint Research Center, Shanghai 200240, China
4 National Engineering Research Center of Light Alloy Net Forming and State Key Laboratory of Metal Matrix Composite, Shanghai Jiao Tong University, Shanghai 200240, China
5 JA Solar Technology Co., Ltd., Beijing 100160, China
* Correspondence: panliu@sjtu.edu.cn

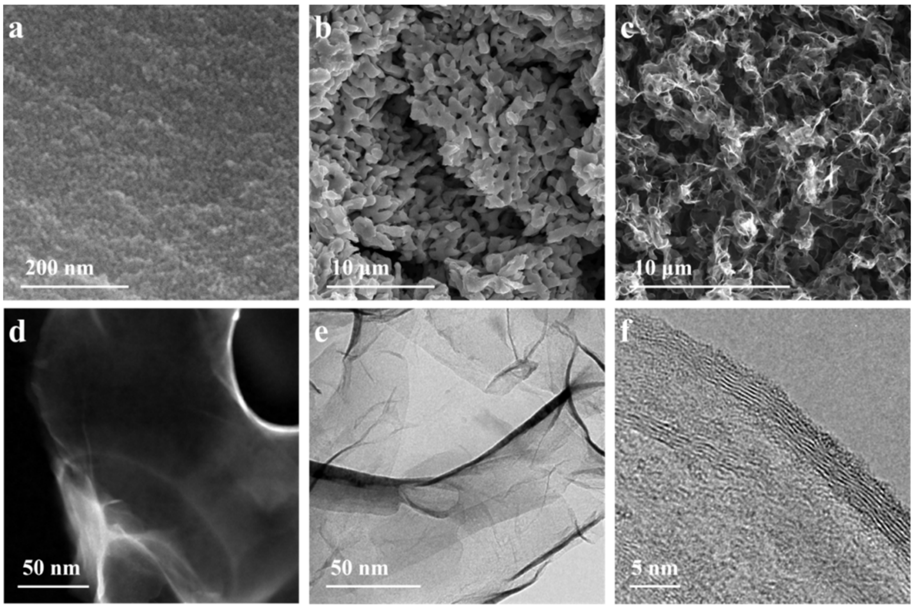

Figure S1. SEM images of np-Ni template (a) before and (b) after growing graphene, (c) np-NG after etching Ni template. (d) STEM and (e) TEM images of np-NG. (f) HRTEM image of the cross-section of np-NG.

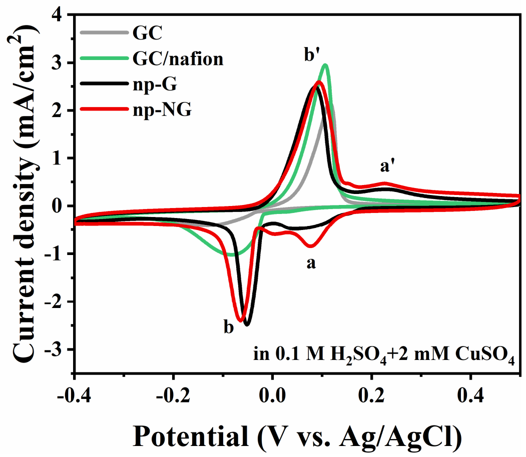

Figure S2. CV curves of different samples in 0.5 M H_2SO_4 containing 2 mM $CuSO_4$.

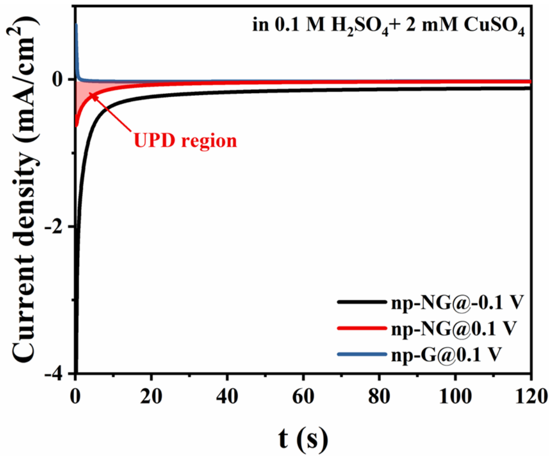

Figure S3. Chronoamperometry curves of the deposition of Cu at different potentials.

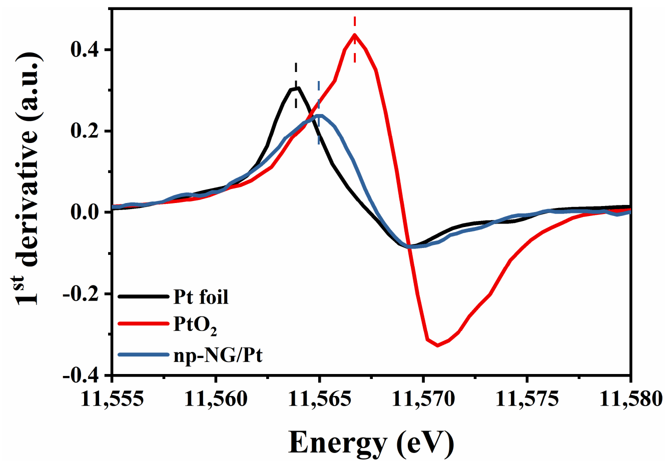

Figure S4. The corresponding first-derivative XANES spectra of different Pt samples.

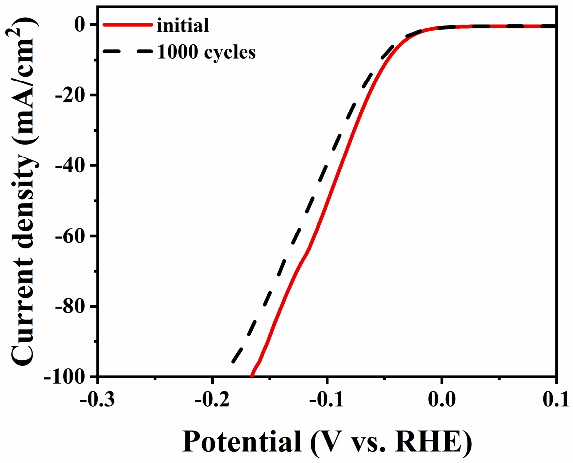

Figure S5. Stability test of np-NG/Pt by potential cycling before and after 1000 cycles.
